# TEMPO functionalized C_60_ fullerene deposited on gold surface for catalytic oxidation of selected alcohols

**DOI:** 10.1007/s11051-017-3857-z

**Published:** 2017-04-27

**Authors:** Piotr Piotrowski, Joanna Pawłowska, Jarosław Grzegorz Sadło, Renata Bilewicz, Andrzej Kaim

**Affiliations:** 1 Department of Chemistry, University of Warsaw, Pasteura 1, 02-093 Warsaw, Poland; 2 Institute of Nuclear Chemistry and Technology, Dorodna 16, 03-195 Warsaw, Poland

**Keywords:** Fullerene, Self-assembly, TEMPO, Catalyst, Alcohol oxidation, Heterogeneous nanostructured catalysts

## Abstract

C_60_TEMPO_10_ catalytic system linked to a microspherical gold support through a covalent S-Au bond was developed. The C_60_TEMPO_10_@Au composite catalyst had a particle size of 0.5–0.8 μm and was covered with the fullerenes derivative of 2.3 nm diameter bearing ten nitroxyl groups; the organic film showed up to 50 nm thickness. The catalytic composite allowed for the oxidation under mild conditions of various primary and secondary alcohols to the corresponding aldehyde and ketone analogues with efficiencies as high as 79–98%, thus giving values typical for homogeneous catalysis, while retaining at the same time all the advantages of heterogeneous catalysis, e.g., easy separation by filtration from the reaction mixture. The catalytic activity of the resulting system was studied by means of high pressure liquid chromatography. A redox mechanism was proposed for the process. In the catalytic cycle of the oxidation process, the TEMPO moiety was continuously regenerated in situ with an applied primary oxidant, for example, O_2_/Fe^3+^ system. The new intermediate composite components and the final catalyst were characterized by various spectroscopic methods and thermogravimetry.

**Electronic supplementary material:**

The online version of this article (doi:10.1007/s11051-017-3857-z) contains supplementary material, which is available to authorized users.

**Graphical abstract:**
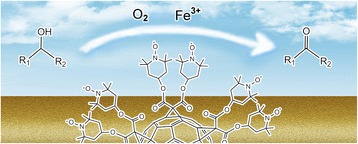
ᅟ

## Introduction

A variety of investigations have revealed that 2,2,6,6-tetramethylpiperidine-1-oxyl (TEMPO) and its derivatives are some of the most efficient catalysts for the selective aerobic oxidation of alcohols to the corresponding carbonyl compounds (de Nooy et al. 1996). The reported catalytic systems are exemplified by both homogeneous (Semmelhack et al. 1984; Greene et al. 2015) and heterogeneous procedures (Bolm and Fey 1999; Yang et al. 2006). It is well-known that both of these procedures have their advantages and drawbacks. High reaction rates, easy removal from the reaction mixture, efficient recycling, low catalyst loading, and simple procedures for the restoration of catalytic activity as part of the reaction’s work-up are among the desirable features required.

Recently, fullerene C_60_TEMPO_*n*_ (*n* = 2, 4, 12) derivatives (Beejapur et al. 2013, 2014) were applied as recyclable catalysts for the oxidation of alcohols through the “release and catch” approach (Gruttadauria et al. 2013). In this strategy, the catalytic system is initially immobilized on a silica multilayer support, but the catalytic moiety is released into the solution over the course of the reaction, and then it is recaptured at the end of the reaction. According to the authors, in this way, valuable combination of the advantages from homogeneous (high catalytic activity and reaction rates) and heterogeneous catalysis (easy separation by filtration) can be achieved. Nonetheless, although the catalytic systems turned out to be highly effective for the oxidation of alcohols, the proposed catalyst recycling procedure still suffered from several restitution steps, such as filtration, solvent removal, and readsorption onto the support.

In contrast, C_60_TEMPO_*n*_ catalytic systems covalently linked to some kind of insoluble support should allow for at least some of the operations to be omitted. We recently reported a procedure for the deposition of in situ deprotected thioacetyl-functionalized C_60_ fullerene derivatives onto gold surface through Au-S bonds. The resulting C_60_ fullerene nanostructured films were then employed as a catalyst and also as an initiator in electrochemical polymerization (Piotrowski et al. 2014, 2015). In the present contribution, we describe the synthesis, characterization and catalytic activity of a novel C_60_ fullerene malonate adduct, functionalized with ten TEMPO radicals and one 8-(acetylthio)octyl substituent, that was subsequently used to decorate the surface of fine gold microspheres. The resulting C_60_TEMPO_10_@Au composite system was then used for the selective oxidation of several classes of alcohols.

## Materials and methods

### Reagents

C_60_ fullerene, 8-bromo-1-octanol, malonyl chloride, 4-hydroxy-TEMPO, iodine, 9,10-dimethylanthracene (DMA), tetrabromomethane, benzyl alcohol, 4-methoxybenzyl alcohol, cyclohexanol, allyl alcohol, diphenylmethanol, 1-phenylethanol, toluene, and *n*-hexane were purchased from Sigma-Aldrich. 1,8-Diazabicyclo[5.4.0]undec-7-ene (DBU), ethyl malonyl chloride, potassium thioacetate, silica gel 70–230 mesh, and gold powder spherical 0.5–0.8 μm S.A. 0.45–0.7 m^2^/g were obtained from Alfa Aesar. Sodium sulfate, acetonitrile, triethylamine, methylene chloride, dimethylformamide, and ethyl acetate were purchased from POCh (Poland). Methylene chloride, dimethylformamide, and toluene were dried and purified before use according to standard procedures. Other solvents were HPLC or analytical grade reagents and were used as received.

### Characterization methods

ESI-MS spectra were acquired on a Micromass LCT ESI-TOF mass spectrometer equipped with an orthogonal electrospray ionization source.

^1^H and ^13^C NMR spectra were recorded on Varian Unity Plus 300- and 500-MHz spectrometers using CDCl_3_ as solvent.

The infrared experiments on fullerene malonates were carried out using a Nicolet 8700 spectrometer while those on C_60_ fullerene derivatives used a Shimadzu FTIR-8400S.

The electron spin resonance (ESR) spectra were recorded under aerobic conditions using a Bruker EMXplus system equipped with an ER 4131 VTM temperature control system. The measurements were performed using ESR quartz tubes with *ϕ* = 4 mm for both the solid state at RT and the 3.24 × 10^−3^ M toluene solution. In the latter case, a 10 K temperature step in the temperature range of 230–360 K was employed; the temperature was stepped up starting from the frozen state at 100 K (larger steps were applied initially). A modulation frequency of 100 kHz and a modulation amplitude of 1 × 10^−4^ mT were applied.

Atomic force microscopy (AFM) measurements were carried out with a nanoScience Instruments Nanosurf easyScan 2 AFM.

XPS measurements were carried out using a VG ESCALAB 210 electron spectrometer equipped with an Al K_α_ source (1486.6 eV). XPS data were calibrated using the binding energy of Au 4f_7/2_ = 84.0 eV as the internal standard.

Thermogravimetric analysis was performed under a high purity nitrogen atmosphere using TA Instruments Q50 Thermal Gravimetric Analyzer with a heating rate of 5 K/min.

Cyclic voltammetry (CV) experiments were carried out using an Autolab potentiostat (ECO Chemie, Netherlands), with a silver/silver chloride (Ag/AgCl) electrode as the reference electrode, platinum foil as the counter electrode, and a the glassy carbon electrode (GCE, BASi, 3 mm diameter) or a modified gold electrode (Gold Arrandee, GmbH) as the working electrode. 0.1 M TBAHFP/toluene/acetonitrile was used as the supporting electrolyte solution. An argon blanket was used to deaerate the solution during the experiments.

High-performance liquid chromatography (HPLC) was performed using a Waters 600E pump, equipped with a Waters 486 tunable absorbance detector or a Waters 410 differential refractometer RI detector, a Gilson FC 203B fraction collector, and a BaseLine Chromtech data system. For isolation of the C_60_ hexakis adduct a Phenogel GPC column (22.5 × 250 mm, 10 μm, 100 Å) with a 5-ml/min flow of toluene was employed. The analysis of the products obtained from the C_60_TEMPO_10_-catalyzed oxidation of selected alcohols was performed using a Phenomenex Luna C_18_ column (4.6 × 250 mm, 5 μm, 50 Å) with a water/acetonitrile mixture as eluent.

### Synthesis

#### 8-Bromooctyl ethyl malonate

A solution of ethyl malonyl chloride (301 mg, 2 mmol) in dry CH_2_Cl_2_ (5 ml) was added dropwise over a period of 15 min to a solution of 8-bromo-1-octanol (418 mg, 2 mmol) and triethylamine (202 mg, 2 mmol) in anhydrous methylene chloride (20 ml) at 0 °C under a nitrogen atmosphere. The resulting mixture was allowed to heat to room temperature and was stirred for additional 4 h. After evaporation of the solvent under reduced pressure, the residue was chromatographed using column chromatography (silica gel 70–230 mesh, ethyl acetate/*n*-hexane 1:6).

Yield, 92%; the mass spectrum (ESI-MS) showed an [M,^79^Br + Na]^+^ peak at 345.2 and an [M,^81^Br + Na]^+^ peak at 347.1 (Fig. S1, Electronic Supplementary Material (ESM)); IR (neat) *ν*_max_(cm^−1^) 2931.6, 2856.4, 1748.2, 1730.1, 1464.8, 1368.4, 1329.3, 1267.5, 1184.4, 1146.1, 1032.1, 726.2, 682.4, 642.4, 604.5, see (Fig. S2, ESM); δ^1^H (500 MHz; CDCl_3_; TMS) 4.18 (q, J = 7.1 Hz, 2H), 4.11 (t, J = 6.7 Hz, 2H), 3.38 (t, J = 6.8 Hz, 2H), 3.34 (s, 2H), 1.88–1.77 (m, 2H), 1.67–1.56 (m, 2H), 1.45–1.37 (m, 2H), 1.31 (m, 6H), and 1.25 (t, J = 7.2 Hz, 3H) ppm (Fig. S3, ESM); δ^13^C (125 MHz; CDCl_3_) 166.51, 65.40, 61.42, 41.55, 33.71, 32.50, 28.85, 28.47, 28.28, 27.91, 25.54, and 13.87 ppm (Fig. S4, ESM).

#### 8-(Acetylthio)octyl ethyl malonate

Potassium thioacetate (171 mg, 1.5 mmol) was added to a stirred solution of 8-bromooctyl ethyl malonate (345 mg, 1 mmol) in dry DMF (15 ml) and stirred for 16 h at room temperature under nitrogen. The reaction mixture was then poured into diethyl ether (100 ml), and a white precipitate formed. After filtration, the solution obtained was washed with water (3 × 10 ml) and subsequently dried over anhydrous sodium sulfate. After filtration and solvent evaporation, the crude product was purified by the means of column chromatography (silica gel 70–230 mesh, ethyl acetate/*n*-hexane 1:4).

Yield, 97%; the mass spectrum (ESI-MS) showed an [M + Na]^+^ peak at 341.2 (Fig. S5, ESM); IR (neat) *ν*_max_(cm^−1^) 2929.9, 2855.8, 1748.2, 1730.1, 1689.2, 1464.8, 1412.0, 1368.3, 1329.6, 1268.2, 1184.3, 1136.1, 1032.3, 954.4, 730.0, 674.2, 624.8, see (Fig. S6, ESM); δ^1^H (500 MHz; CDCl_3_; TMS) 4.21 (q, J = 7.1 Hz, 2H), 4.14 (t, J = 6.7 Hz, 2H), 3.37 (s, 2H), 2.85 (t, J = 6.8 Hz, 2H), 2.32 (s, 3H), 1.67–1.61 (m, 2H), 1.59–1.53 (m, 2H), 1.39–1.25 (m, 8H), and 1.28 (t, J = 7.2 Hz, 3H) ppm (Fig. S7, ESM); δ^13^C (125 MHz; CDCl_3_) 195.64, 166.51, 65.40, 61.42, 41.55, 36.29, 33.71, 32.50, 28.85, 28.47, 28.28, 27.91, 25.54, and 13.87 ppm (Fig. S8, ESM).

#### 61-Ethyloxycarbonyl-61-[8-(acetylthio)octyl-1-oxycarbonyl]-1,2-methano[60]fullerene (I)

The synthesis was performed according to the modified procedure proposed by Bingel (1993). To a solution of C_60_ (144 mg, 0.2 mmol) in freshly distilled toluene (120 ml), a solution of 8-(acetylthio)octyl ethyl malonate (34 mg, 0.1 mmol) in toluene (5 ml), a solution of iodine (25 mg) in toluene (10 ml) and DBU (31 μl, 0.2 mmol) were added. The resulting mixture was stirred at room temperature for 16 h under a nitrogen atmosphere. After concentration using a rotary evaporator, the mixture obtained was chromatographed (silica gel 70–230 mesh, toluene/*n*-hexane 1:1).

Yield, 12%, (the yield was lowered by a side reaction of DBU with the *S*-acetyl group (Singh et al. 2010)); the mass spectrum (ESI-MS) showed an [M + Na]^+^ peak at 1059.4 (Fig. S9, ESM); IR (KBr disk) *ν*_max_(cm^−1^) 2921.9, 2850.5, 1743.3, 1688.4, 1652.6 1458.7, 1427.5, 1266.6, 1253.2, 1233.4, 1205.1, 1186.7, 1132.1, 1094.7, 1060.1, 711.2, 703.0, 668.5, 626.4, 580.3, 552.0, 527.0, see (Fig. S10, ESM); δ^1^H (500 MHz; CDCl_3_; TMS) 4.56 (q, J = 7.1 Hz, 2H), 4.50 (t, J = 6.6 Hz, 2H), 2.86 (t, J = 6.8 Hz, 2H), 2.32 (s, 3H), 1.88–1.78 (m, 2H), 1.54–1.58 (m, 4H), 1.49 (t, J = 7.2 Hz, 3H), and 1.30–1.40 (m, 6H) ppm (Fig. S11, ESM); δ^13^C (125 MHz; CDCl_3_) 195.97, 163.57, 145.32, 145.22, 145.19, 145.3, 145.10, 145.09, 144.81, 144.61, 144.60, 144.58, 144.53, 143.81, 143.02, 143.01, 142.94, 142.92, 142.91, 142.14, 141.84, 140.88, 139.01, 138.86, 71.56, 67.33, 63.36, 30.60, 29.42, 29.02, 28.98, 28.97, 28.65, 28.48, 25.83, and 14.19 ppm (Fig. S12, ESM).

### Hexakis adduct (II)

**Fig. 1:**
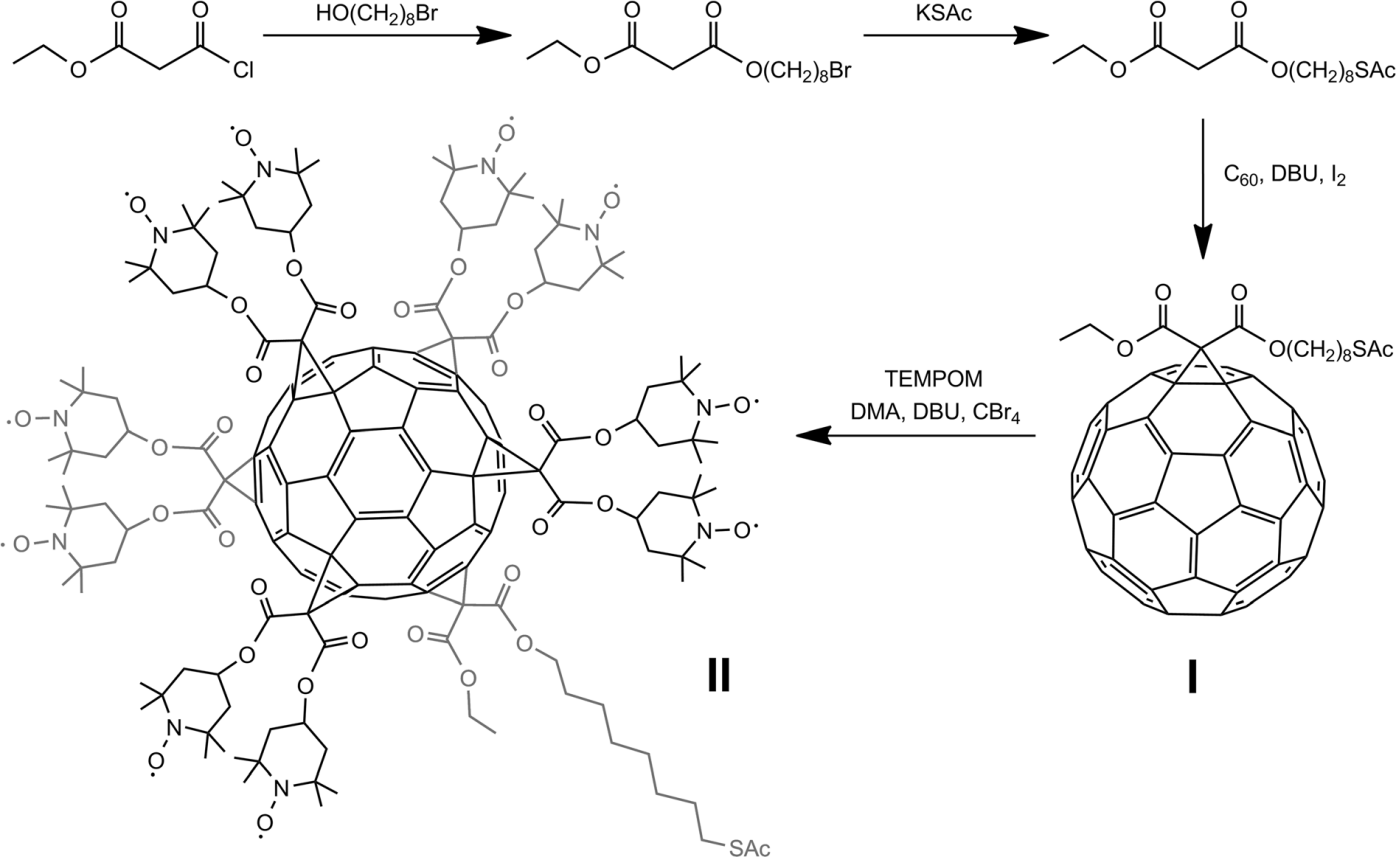
Synthesis scheme for the preparation of C_60_TEMPO_10_ thioacetate

TEMPO malonate (TEMPOM) was synthesized according to methodology reported by Bosch-Navarro et al. (2013). The hexakis adduct was obtained by templated cyclopropanation (Camps and Hirsch 1997) of fullerene thioacetate **I** (Fig. 1). Briefly, a solution of **I** (25 mg, 0.025 mmol) and DMA (51 mg, 0.25 mmol) in dry toluene (6 ml) was stirred at room temperature for 2 h. A mixture of TEMPO malonate (41 mg, 0.25 mmol) and tetrabromomethane (82 mg, 0.25 mmol) in toluene (4 ml) was then added. DBU (38 μl, 0.25 mmol) was then injected and the resulting solution was stirred at room temperature under nitrogen atmosphere for a further 72 h. The desired C_60_TEMPO_10_ thioacetate [5:1] hexakis adduct (II) was isolated from the resulting mixture using an HPLC instrument equipped with a GPC column.

Yield, 53%; the mass spectrum (ESI-MS) showed an [M + Na]^+^ peak at 3112.8 (Fig. S13, ESM); IR (KBr disk) *ν*_max_(cm^−1^) 2974.1, 2936.1, 1747.0, 1689.5, 1464.5, 1379.1, 1364.5, 1220.7, 1178.3, 1115.5, 1078.8, 1004.1, 960.3, 715.2, 528.5, see (Fig. S14, ESM); δ^1^H (500 MHz; CDCl_3_; TMS; after reduction with phenylhydrazine) 5.19–5.24 (m, 20H), 4.30 (q, J = 7.0 Hz, 2H), 4.22 (t, J = 6.7 Hz, 2H), 2.83 (t, J = 6.8 Hz, 2H), 2.32 (s, 3H), 2.00–2.03 (m, 4H), 1.62–1.66 (m, 8H), 1.13–1.31 (m, 120H), and 0.88 (t, J = 7.2 Hz, 3H) ppm (Fig. S15, ESM);

### Self-assembly of the derivative on the gold surface

Experiments without deprotection of *S*-acetyl group did not allow for the production of the desired assembly. For this purpose, the fullerene thioacetate was converted to its thiol analogue using the previously reported in situ procedure (Piotrowski et al. 2014). Self-assembly of the deprotected C_60_TEMPO_10_ was then achieved by dipping the gold substrates into the obtained solution. The resulting films were washed with toluene and dried under a stream of argon.

### Oxidation of alcohols

For oxidation of the selected alcohols, we decided to use the protocol recently reported by Ma et al. (2011), but replacing the TEMPO with the gold microspheres modified with the synthesized nitroxyl C_60_ fullerene derivative. In a typical procedure, to a vigorously stirred mixture of Fe(NO_3_)_3_·9H_2_0 (0.05 mmol), acetonitrile (5 ml), C_60_TEMPO_10_@Au (15 mg), and NaCl (0.1 mmol), the corresponding alcohol (1 mmol) in MeCN (1 ml) was added. The resulting mixture was stirred under aerobic conditions overnight. It was then filtered to remove the catalyst and passed through a short plug of silica gel to remove inorganic salts. The resulting solution was examined using reverse phase HPLC to determine the concentration of the oxidation products.

## Results and discussion

### ESR

**Fig. 2:**
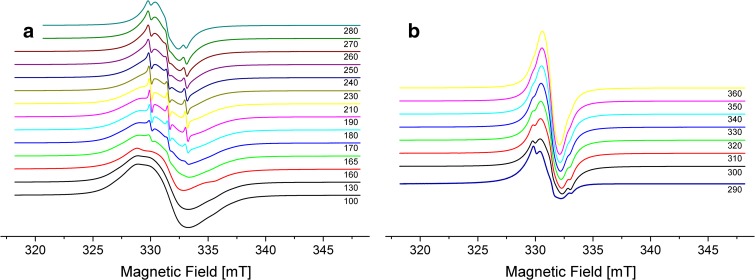
ESR spectra of **II** in toluene in the temperature range **a** 100–280 K and **b** 290-360 K

The ESR spectrum of the C_60_ fullerene–TEMPO adducts in toluene exhibited a triplet centered at *g* = 2.006 with *a*_N_ = 14.98–15.45 G (Ishida et al. 1995; Arena et al. 1997). The interaction between two or more TEMPO nitroxyl radicals has been studied many times, and the corresponding ESR spectra have been described in detail (Porel et al. 2013; Ottaviani et al. 2012). According to the literature data, our ESR experimental results (Fig. 2) confirmed that [5:1]hexakis adduct **II** bore more than one TEMPO radical. For the C_60_TEMPO_10_ thioacetate polynitroxyl, the formal number of lines from the hyperfine interaction was expected (Zeika et al. 2010) to be given by the formula (2*nI* + 1, for ^14^N *I* = 1), giving 21 for *n* = 10, with the intensity of each line following Pascal’s triangle intensities. However, when unpaired electrons are in close proximity the analysis is hampered by the decreasing value of the ^14^N hyperfine splitting value (*a*_N_/*n*) and the large number of lines (Bosman et al. 1997). The problems in resolving ESR multiplets are increased due to the presence of many different transitions from different spin states, arising from multiple interactions between the numerous radical units (Caglieris et al. 2015), and the diversified exchange coupling mechanism, including electron spin-electron spin exchange mediation through bonds and/or through space (Rajca et al. 2006). Nevertheless, in the present C_60_TEMPO_10_ polynitroxide, the EPR spectrum gave direct spectral evidence for the presence of two contributions expressed by a three-line signal, plus a contribution from a broad component, on raising the temperature to ca. 160 K. The former signal, of 2.5 G width, *g* = 2.0059, and *a*_N_ = 15.5 G, clearly corresponds to nitroxide groups that did not interact with any other nitroxide groups of the fullerene–TEMPO10 adduct. The relative intensity of the three lines diminished with temperature, which may have been a result of an increase in the strong spin exchange between the remaining NO groups.

**Fig. 3:**
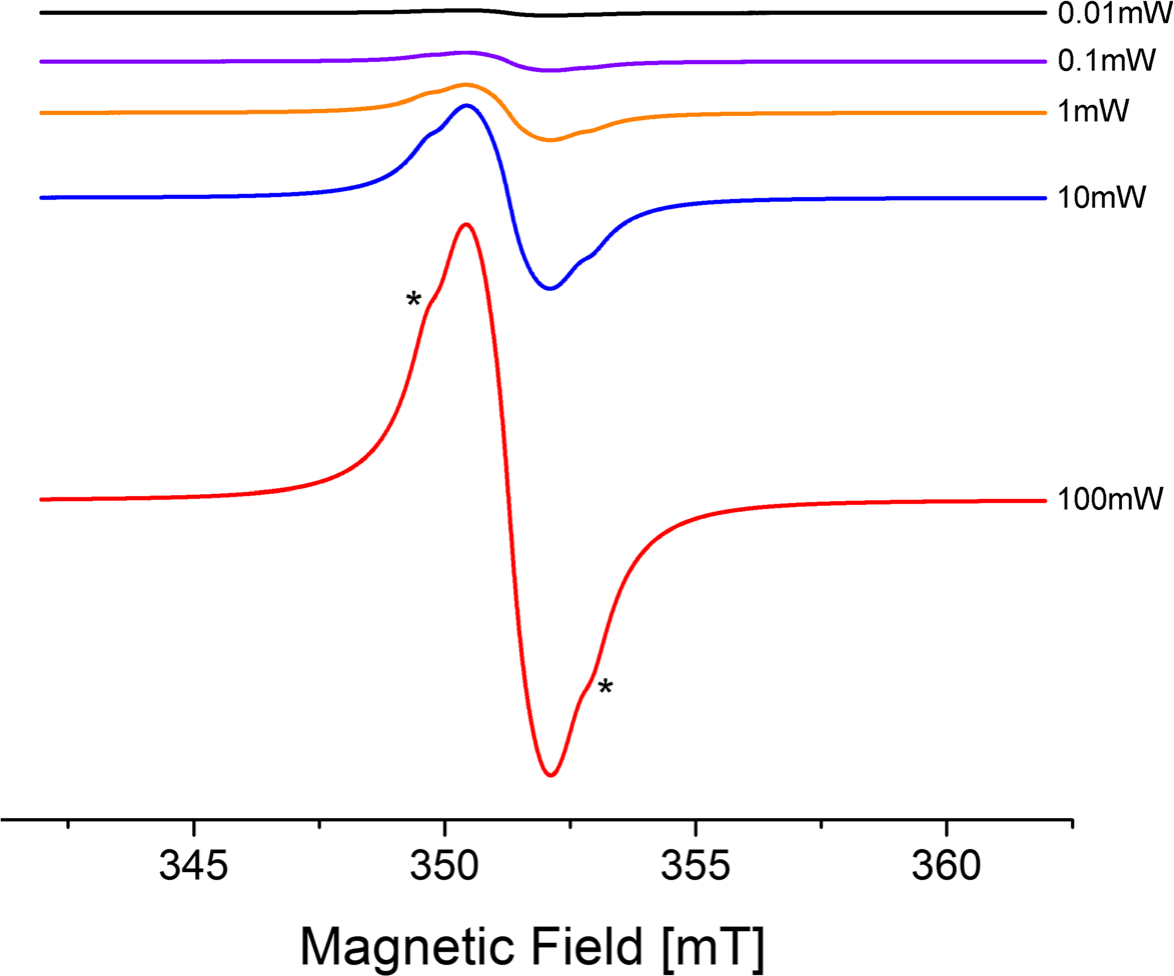
ESR spectra of **II** in the solid state at room temperature. Signal is not saturated till 100 mW

It is also interesting that the main ESR signal (*g* = 2.0066; peak-to-peak splitting ΔHpp = 1.1 G) of **II** in the solid state resembled that of bis(imidazolium)-tagged TEMPO solid catalysts adsorbed on an imidazolium-modified silica gel (Beejapur et al. 2013). In both cases, it was characterized by an additional broad signal (indicated by asterisks), besides the main isotropic singlet (Fig. 3). The presence of the additional signal was probably caused by the presence in the sample of numerous nitroxide fragments, including some not-interacting nitroxide units, since in the case of methanofullerenes substituted with just one or two nitroxide radicals, the corresponding spectra were characterized by singlets only (Gubskaya et al. 2007).

### AFM

**Fig. 4:**
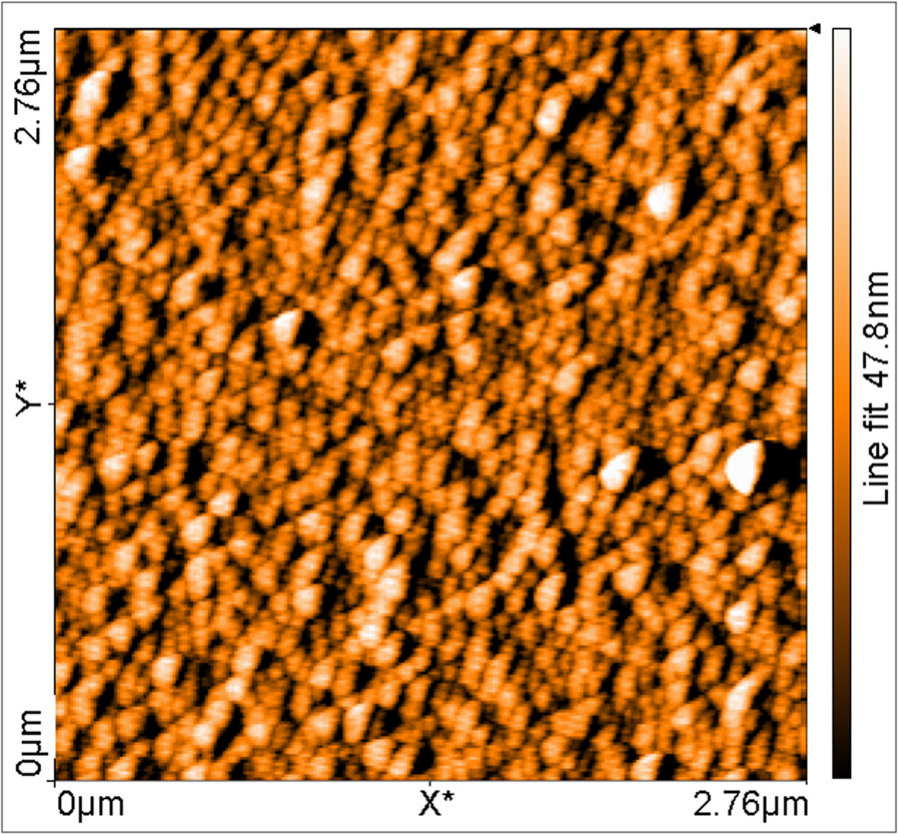
Surface morphology of the C_60_TEMPO_10_ catalyst film on the gold surface obtained by AFM

Atomic force microscopy was used to visualize the C_60_TEMPO_10_ film deposited on the gold surface (Fig. 4). The results obtained showed that immersion of the gold substrate in the solution of the synthesized and in situ deprotected thioacetate lead to the formation of a thick multilayer. In this context, it is worth pointing out that, according to the semi-empirical PM3 calculations (HyperChem™ Professional Release 8.0.8 for Windows Molecular Modeling System, Hypercube, Inc., 1115 NW 4th Street, Gainesville, Florida 32601, USA), the diameter of an optimized C_60_TEMPO_10_ hexakis adduct molecule was estimated to be 2.3 nm. The scanned surface revealed a granular morphology with a roughness height of 47.8 nm, allowing us to conclude that the C_60_TEMPO_10_ derivative had a tendency to assemble on gold as large aggregates rather than a monolayer of single molecules (for the 3D surface visualization see Fig. S16, ESM). This tendency may be advantageous, as it results in a larger number of functionalized fullerenes bound to the gold surface and, consequently, in an enhancement of the active catalytic area.

### XPS

XPS analysis was used to confirm the chemisorption of TEMPO functionalized fullerenes on the gold surface, as well as to investigate the composition of the resulting catalyst. The x-ray photoelectron spectroscopy (XPS) spectrum of the examined nanomaterial showed the presence of gold, sulfur, nitrogen, oxygen, and carbon atoms.

The Au 4f region revealed a double-peak centered at 84.0 and 87.7 eV, corresponding to the Au 4f_7/2_ and Au 4f_5/2_ gold atoms, respectively, typical for the presence of the Au^0^ state (Sashuk and Rogaczewski 2016).

**Fig. 5:**
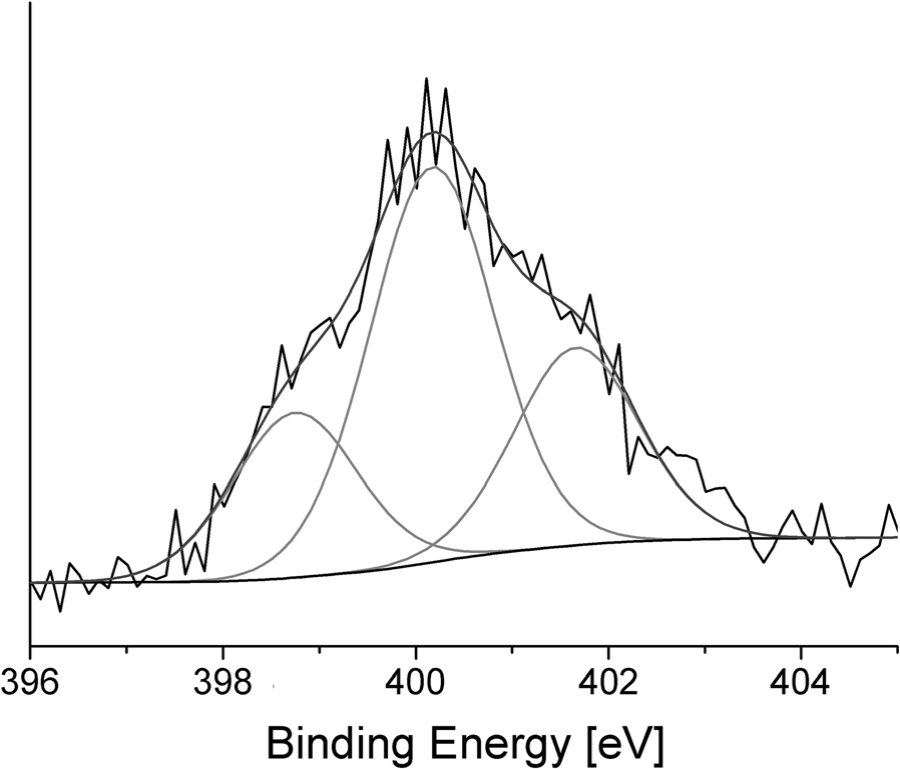
Deconvoluted XPS spectrum in the N1s region of the C_60_TEMPO_10_ sample self-assembled on the Au surface

Results obtained for the N1s region were deconvoluted with very good correlation into three peaks (Fig. 5). The first peak, at 398.7, was related to cyanide anions (Inoue and Fluck 1984), indicating that traces of TBACN remained in the obtained film. The results were in good agreement with the assignment for the highest binding energy peak centered at 401.7 eV, which was observed to be due to the presence of quaternary ammonium nitrogen, from the tetrabutylammonium cation (Srinivasan and Walton 1977; Everhart and Reilley 1981). The atomic ratios determined from the integral intensities of the signals were similar and equal to 21.7 and 26.0% of all the nitrogen atoms. The presence of the remains of a catalyst used for in situ deprotection of an *S*-acetyl group in film formed on a gold surface was recently reported (Piotrowski et al. 2014).

The strongest peak, which was observed at 400.2 eV, can be assigned to nitrogen atoms from nitroxyl radicals according to literature data (Shen et al. 1987). The presence of this signal confirmed successful grafting of the synthesized TEMPO-C_60_ derivative onto gold surface and proved that functionalized fullerenes were the main component of the obtained nanostructured material.

**Fig. 6:**
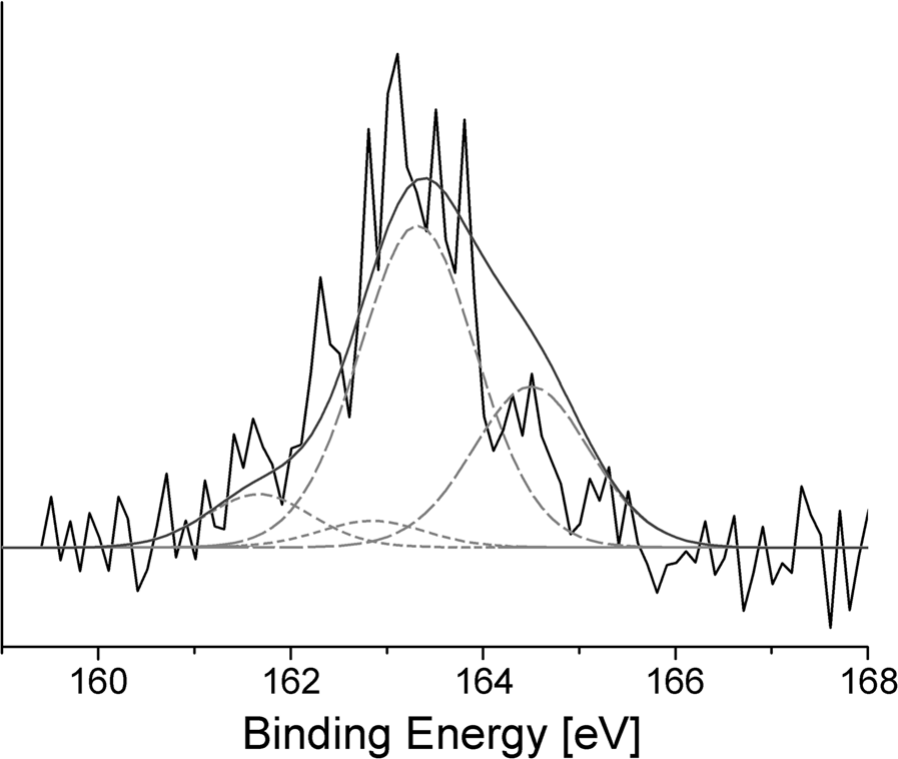
XPS data collected in the S 2p region for C_60_TEMPO_10_ deposited on the gold surface

The unambiguous conclusions from the sulfur S 2p region data were difficult to drawn due to the noisy binding energy curve (Fig. 6). Despite the reservations, the qualitative analysis of the binding energy curve was performed. The resulting fit showed that the signal could be decomposed into two components, with S 2p_3/2_ peaks centered approximately at 161.7 and 163.3 eV, along with their two corresponding S 2p_1/2_ peaks at 162.9 and 164.5 eV, both with the characteristic 2:1 intensity ratio, and the spin-orbit splitting of 1.2 eV (dotted lines on Fig. 6). Photoemission peaks at lower binding energies can be attributed to thiol components bound with the Au surface (Castner et al. 1996), thus confirming the successful grafting of the fullerene derivative using a sulfur anchoring group.

The intensities of the estimated S 2p contributions cannot be taken quantitatively. Nevertheless, they allowed us to conclude that only a small number of sulfur atoms was involved in S-Au bonds, while the main part of registered S 2p spectrum could be attributed to the physisorbed free thiol groups [30], which were incorporated into the multilayer structure of the obtained film. This could be expected, since the AFM results showed that the deposited film was not a single monolayer. Additionally, the absence of signals at higher binding energies (Canitez et al. 2011) suggested that the obtained catalytic film had not undergone any oxidation during handling.

### Thermogravimetry

**Fig. 7:**
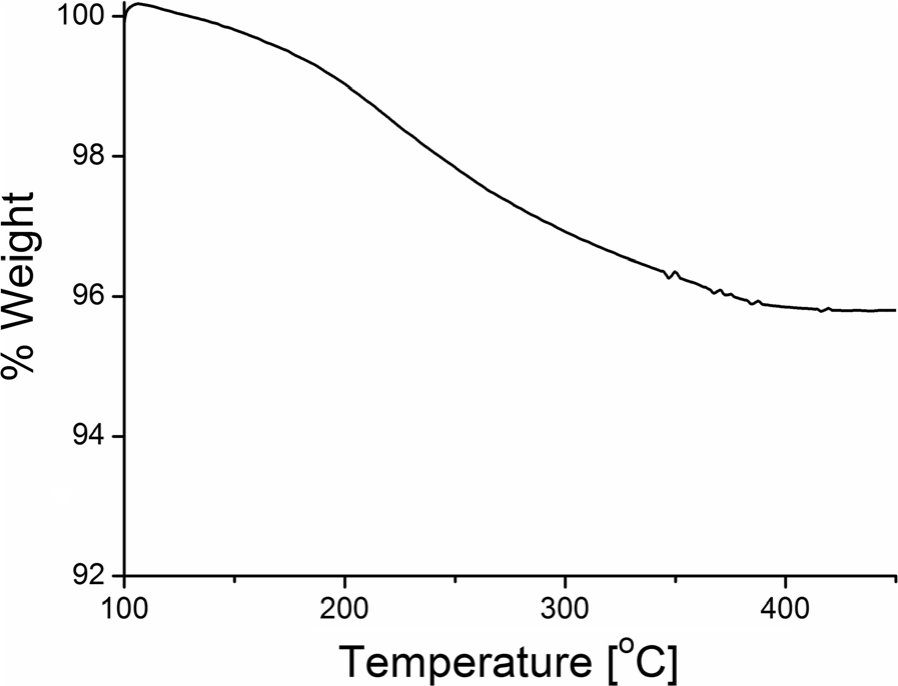
TGA curve of thermal decomposition of C_60_TEMPO_10_@Au catalyst

From the TGA analysis of the deprotected C_60_TEMPO_10_ assembly on the surface of gold the powder (Fig. 7), it was calculated that the nitroxyl functionalized fullerenes constituted around 4 wt.% of the composite with Au. This number was very pleasing, taking into account the size of the gold powder and its density, allowing us to conclude that the Au surface is fully covered with functionalized fullerenes, as seen previously from the AFM imaging. TGA analysis of our catalyst showed that the obtained C_60_TEMPO_10_@Au composite was stable below 100 °C and underwent full thermal decomposition at around 425 °C. These results indicated that the thermal stability was adequate for use, even at moderately elevated temperatures.

### Cyclic voltammetry

**Fig. 8:**
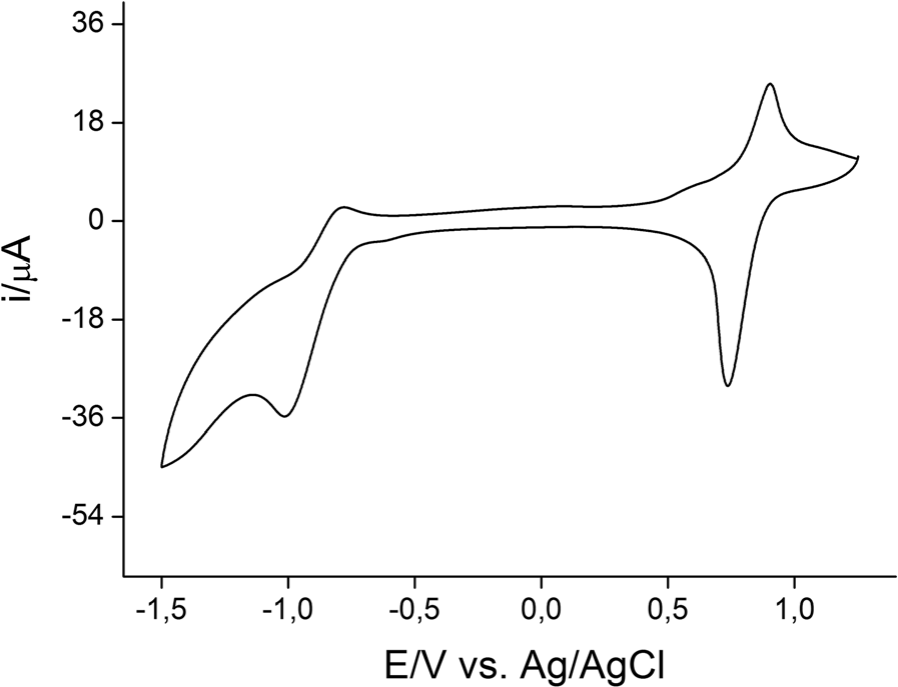
Cyclic voltammogram of TEMPO fullerene **II** in 0.1 M TBAHFP in toluene/acetonitrile (4:1), *v* = 100 mV/s

A cyclic voltammogram obtained for the solution of TEMPO functionalized fullerene thioacetate **II** is shown in Fig. 8. The close to reversible system of redox peaks at 0.81 V is attributed to the TEMPO/TEMPO^+^ redox couple (Krukowski et al. 2009), thus confirming the presence of nitroxyl radicals on the surface of the synthesized C_60_ fullerene derivative. As expected for its hexakis adduct, one main reduction signal is present in the corresponding CV curve (Zhang et al. 2006). This signal was notably shifted toward negative potentials comparing to the values registered for pristine C_60_ fullerene (Piotrowski et al. 2013), allowing us to conclude that **II** has significantly lower electron accepting properties when compared to the unmodified C_60_ core. Additional small peaks could probably be attributed to traces of fullerene derivatives with a lower number of functional groups.

### Catalytic oxidation of selected alcohols

**Fig. 9:**
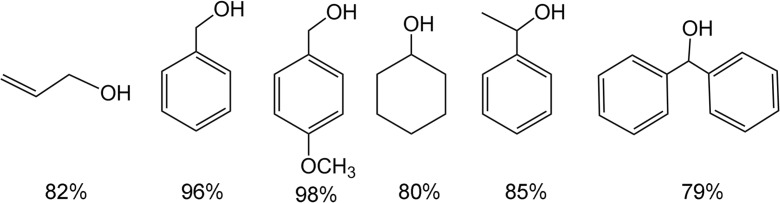
Yields of C_60_TEMPO_10_@Au catalyzed aerobic oxidation of corresponding alcohols (reaction time 16 h)

After the successful chemisorption of the deprotected C_60_TEMPO_10_ thioacetate onto gold powder, which provided a high surface area for the grafting of the nitroxyl fullerene derivative, as well as allowing the removal of the catalytic material by filtration or even decantation of the reaction mixture, the catalyst obtained was employed in the oxidation of various examples of primary and secondary alcohols to their corresponding aldehyde and ketone analogues. The yields of the performed oxidation reactions were determined by reverse phase HPLC and are summarized in Fig. 9, showing the high efficiency of the proposed catalytic system in the oxidation of both primary and secondary alcohols.

**Fig. 10:**
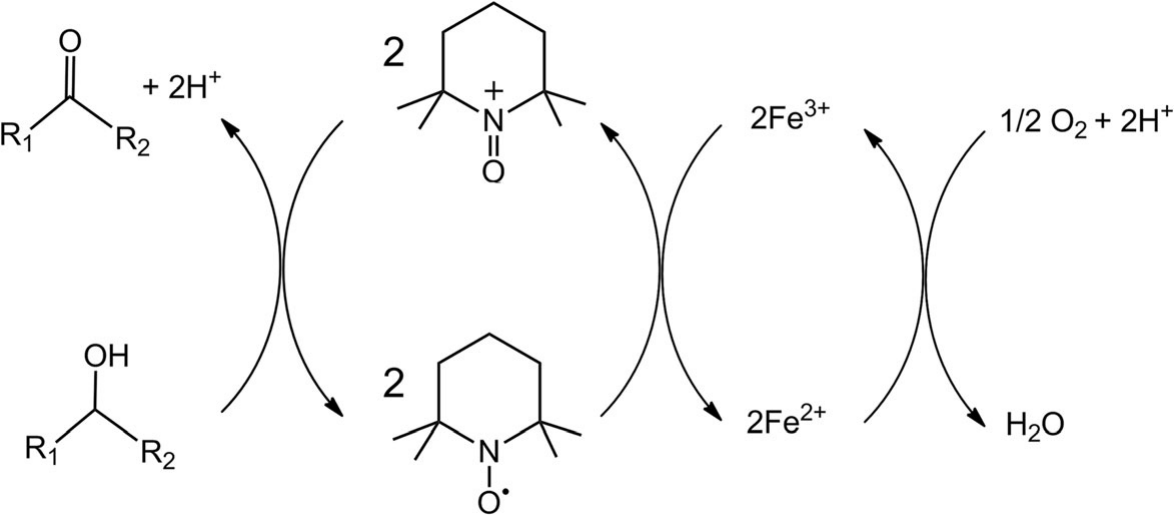
Reaction pathway proposed for the oxidation of alcohols using oxygen and iron (III) nitrate; scheme based on Kim and Jung (2003)

The gold substrate was chosen not only for its high affinity toward thiol species, allowing for the formation of stable assemblies, but also, as a component that, due to its specific interaction with unpaired electrons (Zhang et al. 2003; Alévêque et al. 2009), could reduce the barrier for oxidation (Fig. 10) and thus enhance the activity of the catalyst. This is because the nitroxyl radical undergoes specific interactions with the gold surface, and in this state it might be further oxidized to the oxoammonium cation in the adsorbed state (Krukowski et al. 2009); it therefore transforms to the transient form of TEMPO nitroxyl postulated for the “oxoammonium mechanism” (de Nooy et al. 1996; Adam et al. 2001) for the nitroxyl radical catalyzed alcohol oxidation.

During the process, the TEMPO oxoammonium ion was responsible for the oxidation of alcohols, while being reduced to the hydroxylamine form. To close the catalytic cycle, the TEMPO moiety was continuously regenerated in situ with a primary oxidant, for example, the O_2_/Fe^3+^ system.

## Conclusions

In conclusion, we have designed and synthesized a novel bifunctional C_60_ fullerene derivative, bearing a sulfur anchoring group together with ten nitroxyl radicals. The title compound can be easily self-assembled onto the surface of a microspherical gold support through a covalent S-Au bond, leading to the formation of the efficient alcohol oxidation catalyst C_60_/TEMPO_10_@Au. The catalytic activity of resulting system was evaluated in the oxidation of a variety of primary and secondary alcohols to their corresponding aldehyde and ketone derivatives. The aerobic and room temperature protocol, along with the lack of over-oxidation of primary alcohols to the corresponding carboxylic acids, shows that the presented approach allows the synthesis of aldehyde compounds in excellent yields under mild conditions. Similarly, secondary alcohols are converted into ketone analogues with high yields, even for sterically hindered molecules. The immobilization of the multifunctional C_60_ fullerene derivative on the Au surface allows the removal of catalytic material by simple filtration, while enhancing activity by the simultaneous reduction of the barrier to TEMPO oxidation.

## Electronic supplementary material

ESM 1 (DOCX 915 kb)
