# Supplementary material for: TEMPO functionalized C60 fullerene deposited on gold surface for catalytic oxidation of selected alcohols
Source: J Nanopart Res. 2017 Apr 27;19(5):161. doi: 10.1007/s11051-017-3857-z (PMC5409811; doi:10.1007/s11051-017-3857-z)
Supplement: Supplementary file 1 — (DOCX 915 kb) [file 11051_2017_3857_MOESM1_ESM.docx]

**Supplementary data**

**TEMPO functionalized C_60_ fullerene deposited on gold surface for catalytic oxidation of selected alcohols**

P. Piotrowski,^a^ J. Pawłowska,^a^ J.G. Sadło,^b^ R. Bilewicz^a^ and A. Kaim^a^*

^a^Department of Chemistry, University of Warsaw, Pasteura 1, 02-093 Warsaw, Poland

^b^ Institute of Nuclear Chemistry and Technology, Dorodna 16, 03-195 Warsaw, Poland


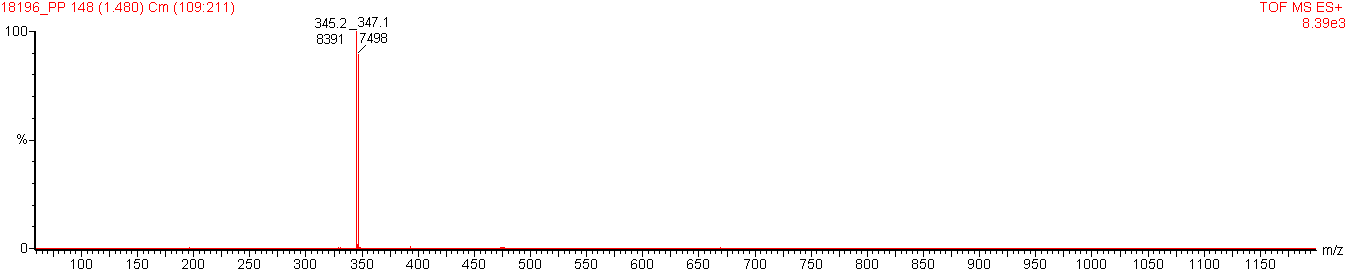


**Figure S1.** ESI-MS spectrum of 8-bromooctyl ethyl malonate.

**
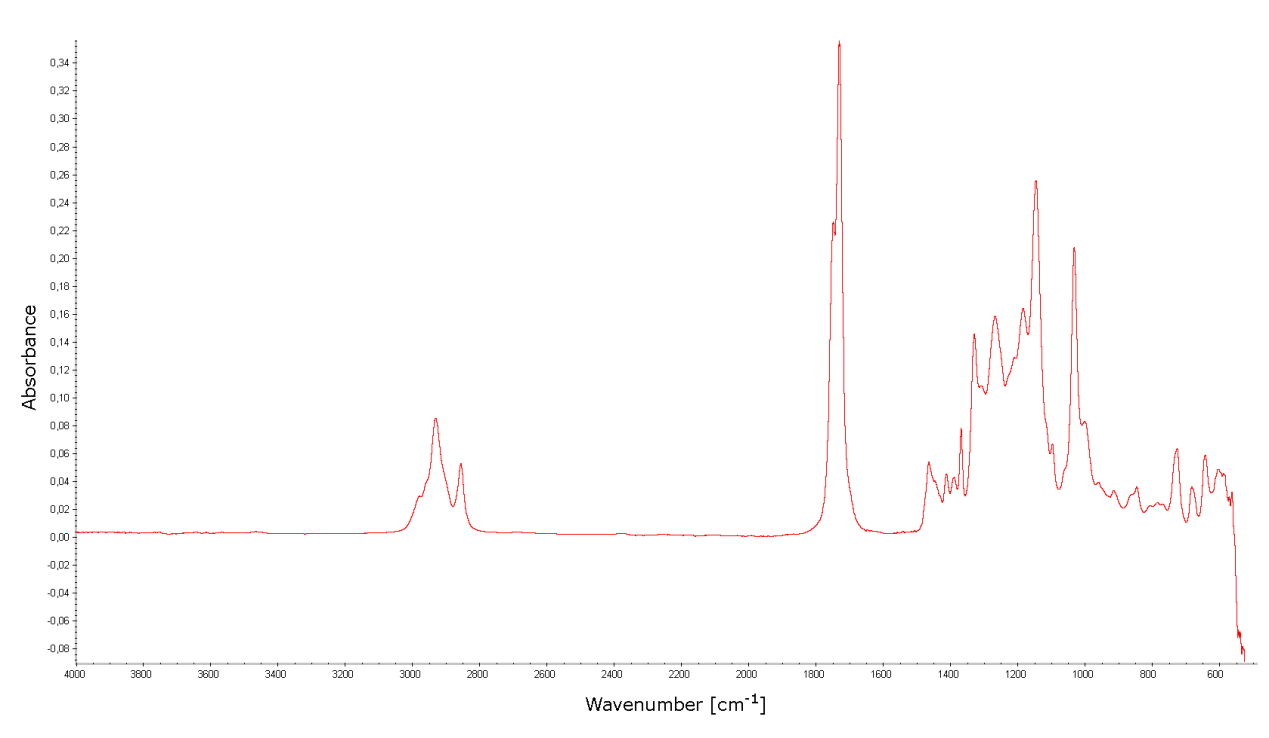
**

**Figure S2.** FT-IR spectrum of 8-bromooctyl ethyl malonate (neat).

**Figure S3.** ^1^H NMR spectrum of 8-bromooctyl ethyl malonate.

**Figure S4.** ^13^C NMR spectrum of 8-bromooctyl ethyl malonate.


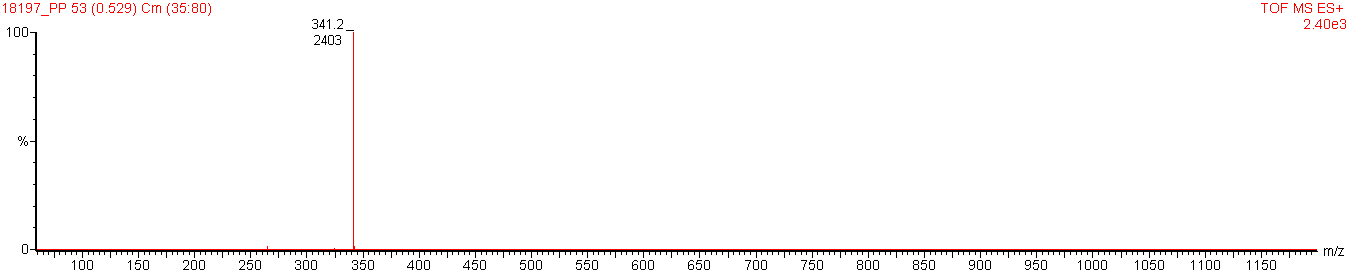


**Figure S5.** ESI-MS spectrum of 8-(acetylthio)octyl ethyl malonate.

**
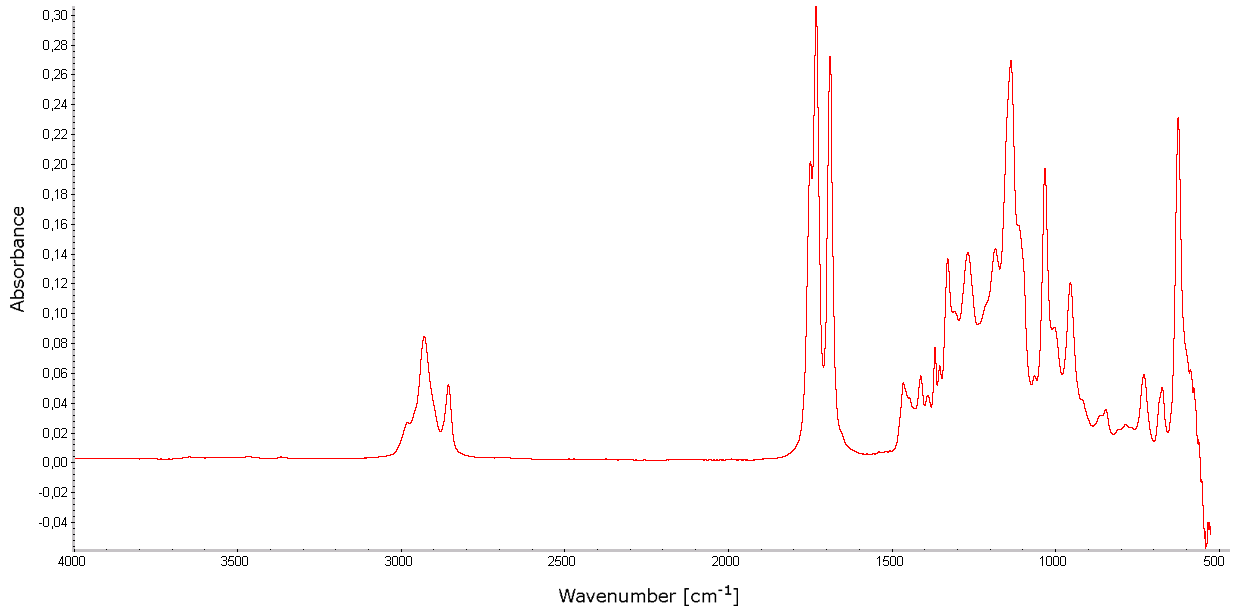
**

**Figure S6.** FT-IR spectrum of 8-(acetylthio)octyl ethyl malonate (neat).

**Figure S7.** ^1^H NMR spectrum of 8-(acetylthio)octyl ethyl malonate.

**Figure S8.** ^13^C NMR spectrum of 8-(acetylthio)octyl ethyl malonate.


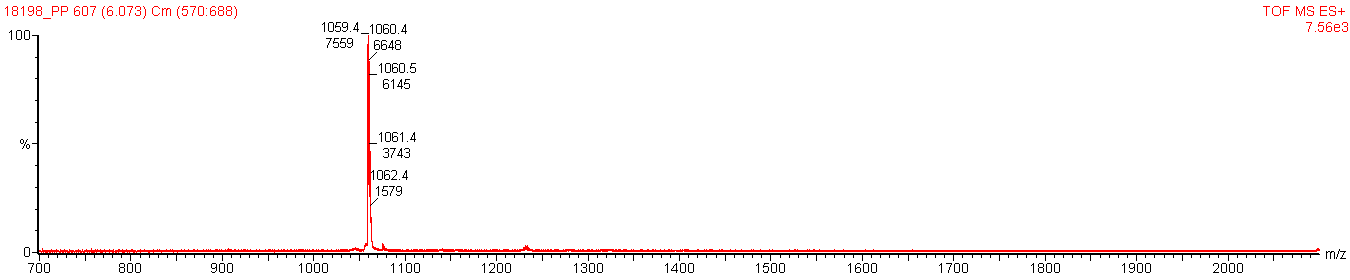


**Figure S9.** Figure S1. ESI-MS spectrum of *S*-acethyl C_60_ fullerene derivative **I.**

**
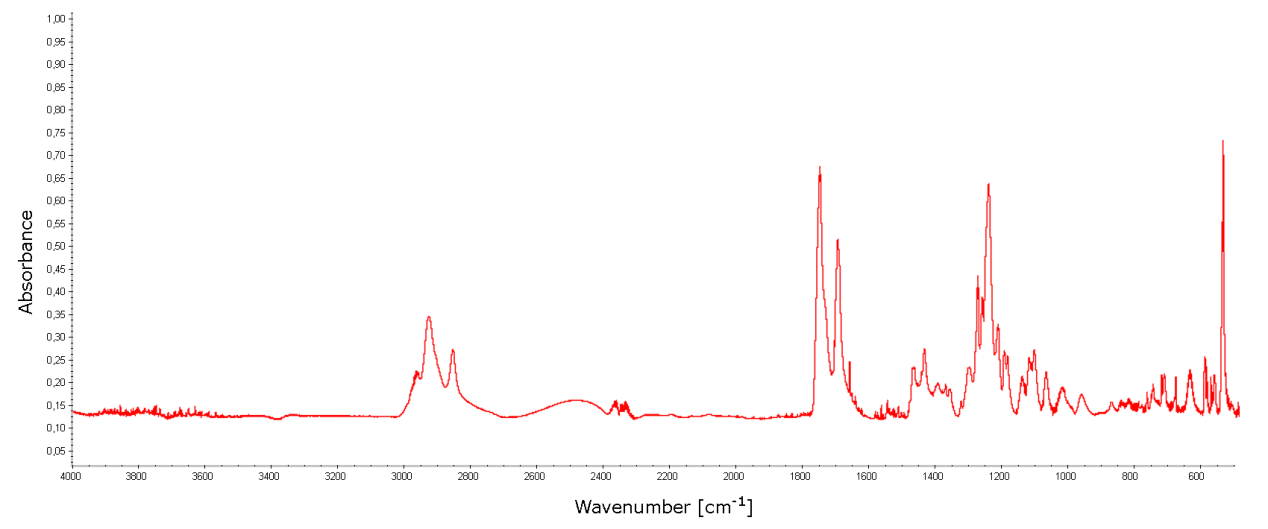
**

**Figure S10.** FT-IR spectrum of *S*-acethyl C_60_ fullerene derivative **I** (KBr disk).

**Figure S11.** ^1^H NMR spectrum of *S*-acethyl C_60_ fullerene derivative **I.**

**Figure S12.** ^13^C NMR spectrum of *S*-acethyl C_60_ fullerene derivative **I.**

**
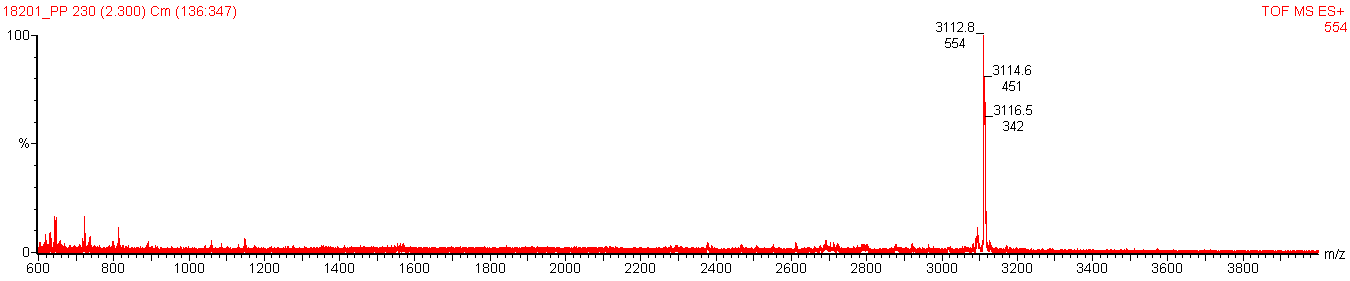
**

**Figure S13.** ESI-MS spectrum of C_60_TEMPO_10_ hexakis adduct **II**.

**
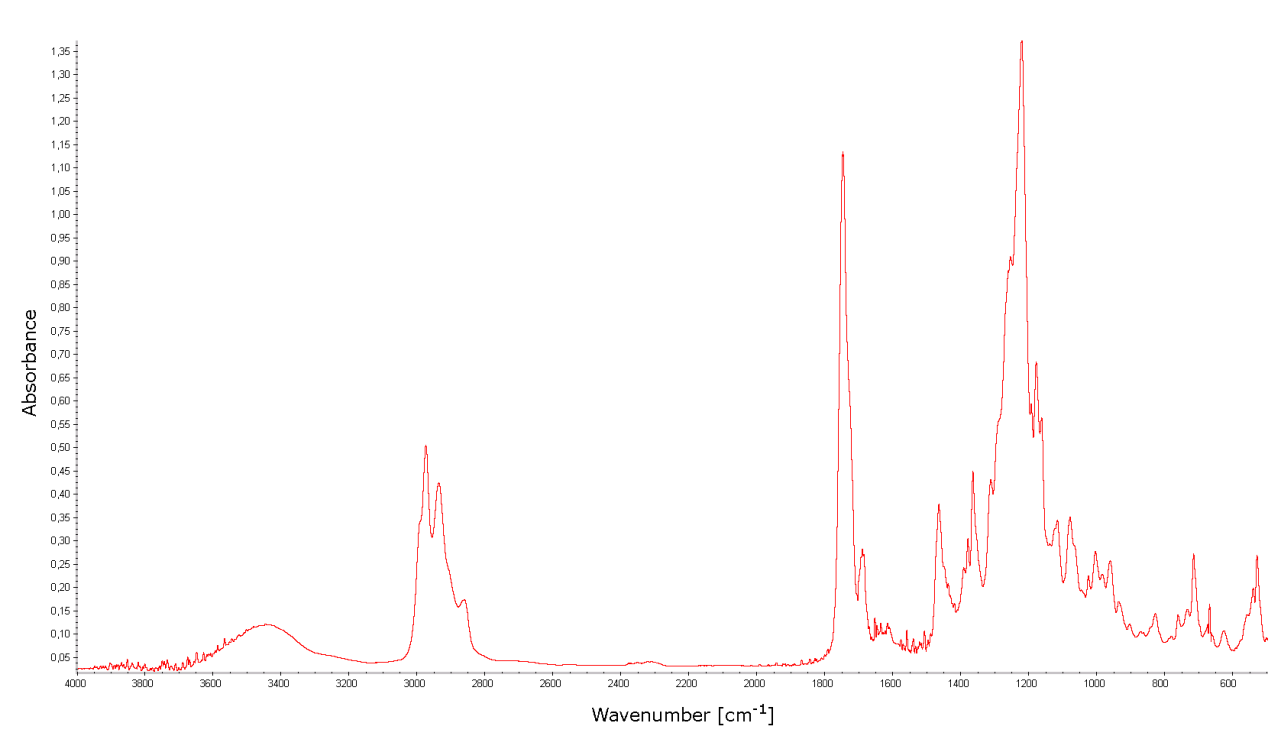
**

**Figure S14.** FT-IR spectrum of C_60_TEMPO_10_ hexakis adduct **II** (KBr disk).

**Figure S15.** ^1^H NMR spectrum of C_60_TEMPO_10_ hexakis adduct **II**.


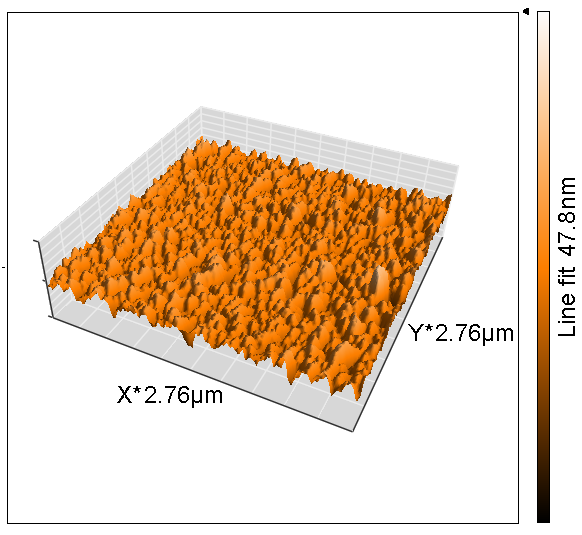


**Figure S16.** 3D surface visualization of the C_60_TEMPO_10_ catalyst film on the gold surface obtained from AFM results (**Fig. 4** in parent manuscript).

­
